# An Observational Study of the Impact of COVID-19 and the Transition to Telehealth on Community Mental Health Center Providers

**DOI:** 10.21203/rs.3.rs-48767/v1

**Published:** 2020-07-29

**Authors:** Marisa Sklar, Kendal Reeder, Kristine Carandang, Mark G Ehrhart, Gregory A Aarons

**Affiliations:** University of California San Diego; University of California San Diego; University of California San Diego; University of Central Florida; University of California San Diego

**Keywords:** COVID-19, telehealth, community mental health, provider perspectives, EBP implementation

## Abstract

**Background:**

The COVID-19 pandemic has swiftly and remarkably altered community mental health service delivery and evidence-based practice (EBP) implementation. This study reports provider perspectives on the impact that COVID-19 had on their work and EBP implementation.

**Methods:**

Providers (*n* = 93) completed online surveys with quantitative measures and open-ended items targeting their responses and/or reactions to COVID-19, and to the transition to providing services via telehealth.

**Results:**

Perceptions of personal risk and rumination around COVID-19 were low, while telehealth was viewed positively by providers. Three major themes emerged regarding the major impacts of COVID-19 on work: 1) the altered nature of interactions between patient/client and provider, 2) changes in provider expectations regarding productivity, and 3) challenges maintaining work-life balance. In regard to the major impacts of COVID-19 on EBP implementation, three themes emerged: 1) increased difficulty delivering certain therapies via telehealth, 2) potential limitations to session confidentiality, and 3) challenge of engaging children in telehealth.

**Conclusions:**

In the context of the COVID-19 pandemic, community mental health providers continued to engage with clients and implement EBPs while navigating a number of changes related to the transition to telehealth. This study highlights the need for further work on what supports providers need to effectively engage with clients and deliver EBPs via telehealth and has implications for how telehealth is sustained or de-implemented in response to COVID-19.

## Background

The 2019 outbreak of the novel coronavirus disease (2019-nCoV/COVID-19) has drastically impacted the context in which mental health services are provided and the context in which evidence-based practice (EBP) implementation is occurring. On March 23, the Governor of Indiana issued a ‘stay at home’ order mimicking restrictions set across the country; among these, gatherings of 10 + people were prohibited and businesses were to conduct their work from home, if possible [1]. In response, many community mental health centers (CMHCs) rapidly integrated telehealth services (i.e., services provided via phone and/or video platforms) to minimize the risk of COVID-19 transmission to their clients, staff, and the general public [2]. Nationally, HIPAA requirements, and Medicare/Medicaid billing requirements, were adjusted to support this transition [3–6]. Mental health providers were subsequently tasked with transitioning implementation of EBPs from in-person treatment to telehealth. In accordance with the STROBE checklist of items included in reports of observational studies (Additional file 1), this paper reports on the impact that the COVID-19 pandemic had on providing mental health services and implementing EBPs from the perspectives of CMHC providers in Indiana.

## Methods

### Participants and Setting

Data were collected as part of an ongoing service contract between the UC San Diego team and the Indiana Division of Mental Health and Addictions (DMHA) to engage in the Leadership and Organizational Change for Implementation (LOCI) strategy to facilitate an implementation climate for evidence-based practice (EBP) in Indiana CMHCs [7–9]. Participants were providers (n = 93) from 6 CMHCs that are also contracted with Indiana DMHA to improve upon implementation of combined motivational enhancement therapy and cognitive behavioral therapy (MET/CBT) and other EBPs. Providers identified mostly as female (*n* = 77; 84.6%), non-Hispanic (*n* = 81; 89%), White (*n* = 79; 86.8%), and were 41 years old on average (*sd*= 14.8 years). A majority of providers had completed master’s level education (*n*= 65; 71.4%) and identified social work as their primary discipline (*n* = 44; 48.4%). On average, providers reported having worked with their present agencies for 4.7 years (*sd*= 7.7 years), and being in their current positions for 3.2 years (*sd*= 5.9 years). Providers reported spending the greatest percentage of their work time in psychotherapy and/or counseling ($\bar{x}=44.1\%$) and reported an average caseload of 52.1 (*sd*= 39.5) clients per month. Administrative work (e.g., documentation, billing) ($\bar{x}=18.1\%$) and case management ($\bar{x}=12.0\%$) comprised the next greatest percentages of providers’ work time. See Table 1 for more information regarding provider demographics.

### Procedures And Measures

CMHC providers completed surveys via the Qualtrics web-based platform that included measures targeting their reactions to the COVID-19 outbreak, and transition to providing services via telehealth. The measures that were utilized are described below. When appropriate, the internal consistency of each measure was assessed for this sample using Cronbach’s a and is included in the description of each measure.

#### Perceptions of Personal Risk.

This 9-item measure was adapted from Wu et al.’s measure assessing perceptions of personal risk around SARS [10]. Items were adapted to assess participants perceived risk of being exposed to, and getting infected with, COVID-19. Response options were also adapted such that participants responded using a 5-point response scale ranging from 0 = “Strongly Disagree” to 4 = “Strongly Agree.” Internal consistency for this measure was high at *a* = .87.

#### COVID-19 Rumination.

This 3-item measure was developed by LeNoble and colleagues to explore the extent to which participants’ rumination about COVID-19 is interfering with their work [11]. Participants responded using a 5-point response scale ranging from 0 = “Strongly Disagree” to 4 = “Strongly Agree.” Internal consistency for this measure was high at *a* = .81.

#### Work Changes due to COVID-19.

These three items were developed by the study authors through an iterative process of item generation, discussion, and refinement until consensus on item wording was achieved. The resulting three items assess the changes in tasks, settings, and teams that mental health providers experienced following the COVID-19 outbreak. Participants responded using a 5-point response scale ranging from 0 = “Not at all” to 4 = “Very great extent.” These items were developed to measure different types of changes that providers may experience, and not an underlying construct of work changes. Combining item responses into a single scale was not appropriate, and as such, internal consistency was not assessed. Items were analyzed individually to better understand the impact of COVID-19 on each individual type of change.

#### Burnout.

The Copenhagen Work Burnout Inventory is 3-item measure developed to assess the extent to which participants have experienced emotional exhaustion and work-related frustration within the past two weeks [12]. Participants responded using a 5-point scale ranging from 0 = “Never” to 4 = “Always.” Internal consistency for this measure was high at *a* = .91.

#### Perceived Organizational Support.

The 3-item perceived organizational support scale was developed to assess the extent to which respondents believe help is available to them from their agency, that their agency cares about their well-being, and their agency shows concern for them [13]. Participants responded using a 5-point response scale ranging from 1 = “Strongly Disagree” to 5 = “Strongly Agree.” Internal consistency for this measure was high at *a* = .93.

#### Telehealth Self-Efficacy.

This 4-item measure was adapted from a measure developed by Lau and Brookman-Frazee to assess participant’s confidence, knowledge, understanding, and preparation to deliver therapy via telehealth [14, 15]. Participants responded using a 5-point response scale ranging from 0 = “Strongly Disagree” to 4 = “Strongly Agree.” Internal consistency for this measure was high at *a* = .92.

#### Collective Efficacy.

This 3-item measure was adapted from Jex and Bliese to assess efficacy beliefs targeting the agency’s transition to telehealth [16]. Participants responded using a 5-point response scale ranging from 0 = “Strongly Disagree” to 4 = “Strongly Agree.” Internal consistency for this measure was moderately high at *a* = .76.

#### Telehealth Beliefs.

This 5-item measure was adapted from the University of Michigan’s Behavioral health Workforce Research Center to assess beliefs regarding the importance of telehealth [17]. Participants responded using a 5-point response scale ranging from 0 = “Strongly Disagree” to 4 = “Strongly Agree.” Internal consistency for this measure was high at *a* = .85.

#### Transition to Telehealth.

Seven items evaluating the transition to telehealth were developed by the study authors through an iterative process of item generation, discussion, and refinement until consensus on item wording was achieved. These items asked participants to indicate the extent to which different aspects of treatment were better or worse when serving clients via telehealth as opposed to in-person treatment; see Table 4 for the individual items. Participants responded using a 5-point response scale ranging from 0 = “Significantly worse with telehealth relative to in-person” to 5 = “Significantly better with telehealth relative to in-person.” Internal consistency for this measure was high at *a* = .82.

#### Open-ended survey questions.

Participants also responded to two open-ended survey items to explore the impacts of COVID-19. The first asked “What have been the major impacts of COVID-19 on your work?” The second asked “What have been the major impacts of COVID-19 on the implementation of MET/CBT and other EBPs?”

### Analysis

Participant responses were aggregated across all items to obtain an overall scale mean with the exception of the Work Changes and the Transition to Telehealth items, which were analyzed individually. Descriptive statistics of quantitative measures were assessed to explore providers’ responses and/or reactions to the COVID-19 outbreak, and subsequent transition to providing services via telehealth. Potential between agency differences in provider responses were explored using univariate analysis of variance (UNIANOVA). Provider responses to open-ended survey questions were first reviewed by authors (MS, KR, and KC) to gain familiarity with the content and to isolate broad themes. Text was then sorted and organized in accordance with broad themes, and new themes were generated when appropriate. All authors held meetings to review “chunks” [18] of text and develop summaries of findings.

## Results

### Quantitative Survey Item and Scale Descriptive Statistics

See Tables 2, 3, and 4 for survey item and scale descriptive statistics. On average, provider item and scale scores were lowest on the COVID-19 Rumination ($\bar{x}=1.10$), and Perceptions of Personal Risk ($\bar{x}=1.41$). Reciprocally, provider item and scale scores were greatest on the Telehealth Beliefs scale ($\bar{x}=2.89$) indicating generally favorable beliefs and attitudes about the importance of telehealth, followed by telehealth self-efficacy ($\bar{x}=2.68$) and perceived organizational support ($\bar{x}=2.66$). With regard to the work changes (Table 2), the highest scores were for changes in the work setting ($\bar{x}=3.66$), followed by changes in the work tasks ($\bar{x}=2.91$). Fewer changes were reported for the work team ($\bar{x}=2.11$). With regard to the questions evaluating the effects of telehealth (Table 4), providers reported the largest benefits for scheduling ($\bar{x}=3.12$), and the biggest challenge with patient/client focus ($\bar{x}=2.38$). Results from UNIANOVA indicated no significant differences between agencies in any of the survey items and/or scale scores (see Table 2 for UNIANOVA).

### Qualitative Open-ended Survey Responses

#### Major impacts of COVID-19 on work.

Analysis of open-ended question responses identified three major themes. One centered on the impact that the transition to telehealth had on patient/client and provider interactions. Providers described technological barriers to high quality interactions identifying challenges such as “blocked cell number,” “caseload lives in rural areas…not all kids have access to internet or stable internet,” and “some clients do not have technological capacity for video conferencing.” Some providers commented on the challenge of developing/maintaining rapport through the transition to telehealth. For example, providers reported “not being able to build rapport with new clients,” and that “not being able to provide therapy in person and be able to read client’s body language has been the major impact.” Some providers reported beliefs that telehealth facilitated improvements in communication with clients. For example, providers stated that “it has been more enjoyable regarding relationships with clients…due to the less formal atmosphere as clients are more comfortable in their homes,” and that they experienced “stronger communication” with clients.

Another theme centered on the changes in provider expectations regarding productivity. Some providers commented on reduced productivity due to this transition, stating “my productivity has dropped as a result because I have been having issues coordinating with some families,” and “I used to have very high guardian engagement.this has decreased; guardians have limited to no access to technology at times.” Some providers commented on increased demands like “increase in documentation required,” “increased pressure regarding productivity and revenue,” and “more new changes in documentation and more paperwork without receiving productivity for increased time spent.”

A third theme emerged and centered on challenges providers reported with maintaining a work-life balance. For example, providers stated that “work-life balance has been disrupted as I have difficulty separating myself from work,” and “lack of home life/work balance.time management is difficult.” Providers also reported challenges with working from home while caring for their children, stating “working from home while taking care of my child makes it difficult to do my best work with clients,” and “it can be difficult to work from home with children.”

#### Major impacts of COVID-19 on EBP implementation.

When asked about the major impacts of COVID-19 on implementation of EBPs, three themes emerged. One centered on the challenge delivering certain therapies via telehealth wherein providers stated “barriers to implementing services like play therapy are somewhat dependent on the setting,” “prize draws and drug screens are difficult to do,” “lack of client ability to access the worksheets or use a video format,” and that they “haven’t tried [Eye Movement Desensitization and Reprocessing]-bilateral stimulation” via telehealth.

A second theme centered on the potential limitations to confidentiality and/or lack of privacy when providing treatment with telehealth. Providers reported challenges engaging clients in EBP for the treatment of trauma because some clients are less willing to discuss traumas due to limited confidentiality/lack of privacy. Some providers stated “many clients now have less privacy at home-may have partners/kids around;” other providers reported discomfort processing trauma without being able to see how their clients are responding stating that “I have not been able to work with some of my patients on healing their trauma…I am uncomfortable due to not being able to see if they are upset, being triggered, etc.”

The final theme that emerged centered on the general challenge of engaging children in treatment upon transitioning to telehealth. Providers stated “it is difficult to teach my students over the video sessions at times depending on the subject we are discussing and distractions,” “some parents don’t hold their children accountable for doing video sessions,” “parents prefer I work with their children face-to-face,” and that “younger kids often engage better face to face.”

## Discussion

This study investigated major impacts of COVID-19 on CMHC providers’ work and EBP implementation. One key impact was the rapid transition to providing services via telehealth. Providers indicated that telehealth was critical to the viability of their organizations. In general, providers viewed their agencies’ transitions to telehealth positively and reported confidence in their abilities to deliver services via telehealth. Although survey results suggested that providers perceived both their therapeutic relationships and their clients’ willingness to schedule telehealth sessions as somewhat better than in-person services, some providers reported difficulty maintaining client engagement and using EBPs in telehealth sessions. Providers also reported experiencing stress related to billing, documentation, and productivity demands while adapting to work changes and maintaining work-life balance.

Providers’ generally favorable views of telehealth is important for mental health agency- and system-level leaders to consider when making decisions on sustained use of telehealth after the pandemic. A number of factors aided the rapid transition to telehealth, including adjusted HIPAA restrictions and Medicaid billing requirements [3–6]. Continued use of telehealth in the US will likely be a function of billing capabilities and other policies to facilitate sustainment. Furthermore, our results suggest that agencies generally did not offer thorough training for telehealth. Should telehealth persist, it is essential for leadership to consider what supports providers need to effectively engage with clients and deliver EBPs via telehealth.

This study lends important insight into provider experiences with telehealth and EBP implementation in the context of the COVID-19 outbreak. There are, however, a number of limitations. Due to the novel nature of this outbreak and the related work changes, some of the administered measures were created or adapted for this study and do not yet have published psychometrics, making cross-study comparison challenging. Also, this study did not include client- or treatment-level information, such as client perspectives on telehealth. This information will be crucial as mental health agencies begin to plan for telehealth de-implementation or sustainment.

## Conclusion

In the context of the COVID-19 outbreak, CMHC providers continued to engage with clients, provide services, and implement EBPs through telehealth. Persistence was needed to connect and engage with clients, and creativity was crucial to continue implementing EBPs. Although telehealth was viewed positively by providers and may be a promising strategy for increasing provider/client communication, it remains unclear how telehealth impacts EBP implementation and client engagement in treatment.

## Supplementary Material

Supplement

## Figures and Tables

**Table 1:** Provider demographics.

−	41.0 ±14.8	
Age (years; *x*±sd)		
Gender	n	%
Female	77	82.8
Male	13	14.0
Other	1	1.1
Missing	2	2.2
Race	n	%
White	79	84.9
Black or African American	3	3.2
Asian	2	2.2
American Indian/Alaska Native	1	1.1
More than one race	6	6.5
Missing	2	2.2
Ethnicity	n	%
Non-Hispanic	81	87.1
Hispanic	10	10.8
Missing	2	2.2
Highest level of Education	n	%
Some college	1	1.1
College graduate	14	15.1
Some graduate work	5	5.4
Master’s degree	65	69.9
PhD, MD, or equivalent	6	6.5
Missing	2	2.2
Primary Discipline	n	%
Drug/Alcohol Counseling	13	14.0
Social Work	44	47.3
Child Development	2	2.2
Marriage and Family Therapy	2	2.2
Psychology	16	17.2
Other	14	15.1
Missing	2	2.2
Providers per Agency	n	%
Agency 1	7	7.9
Agency 2	15	16.9
Agency 3	11	12.4
Agency 4	11	12.4
Agency 5	43	48.3
Agency 6	2	2.2
−	4.7 ±7.7	
**Years at present agency (*x*±sd)**		
−	3.2 ±5.9	
**Years in present position (*x*±sd)**		
Percentage of your work time doing the following…	*x*±sd	
	−	
Standardized assessments	7.1 ±10.6	
Case management	12.0 ± 18.6	
Psychotherapy and/or counseling	44.1 ±28.6	
Administrative work (e.g., documentation, billing)	18.1 ±11.9	
Meeting with your supervisor	7.2 ±10.6	
Supervising others	4.3 ±13.4	
Travel	2.1 ±5.0	
Other	5.1 ±14.5	

**Table 2:** Survey scale descriptive statistics.

	Minimum	Maximum	Mean	Std. Deviation	Test of Between-Agency Differences
Perceptions of Personal Risk	0.00	4.00	1.41	0.83	*F*(5,83) = .89, *p* = .492
COVID-19 Rumination	0.00	3.33	1.10	0.87	*F*(5,83) = 1.13, *p* = .352
Burnout	0.00	4.00	2.31	0.97	*F*(5,83) = 1.84, *p* = .114
Perceived Organizational Support	0.00	4.00	2.66	1.00	*F*(5,83) = .86, *p* = .509
Telehealth Self-Efficacy	0.00	4.00	2.68	0.85	*F*(5,83) = .99, *p* = .426
Collective Efficacy	0.67	4.00	2.55	0.77	*F*(5,83) = .90, *p* = .483
Telehealth Beliefs	0.00	4.00	2.89	0.84	*F*(5,83) = 2.22, *p* = .060

**Table 3:** Work changes due to COVID-19 descriptive statistics.

	Minimum	Maximum	Mean	Std. Deviation
Because of COVID-19, my work tasks have changed	0	4	2.91	1.09
Because of COVID-19, my work setting has changed.	1	4	3.66	0.64
Because of COVID-19, my work team has changed.	0	4	2.11	1.45

**Table 4:** Transition to telehealth descriptive statistics.

	Minimum	Maximum	Mean	Std. Deviation
Relationships between you and your patients/clients.	1	5	2.80	0.71
Quality of communication between you and your patients/clients.	1	5	2.57	0.81
Rate of no-shows with fewer being better.	1	5	2.81	1.22
Patient/client focus during sessions.	1	5	2.38	0.85
Patient/client engagement in treatment.	1	5	2.71	0.88
Confidentiality of discussions with patients/clients.	1	5	2.82	0.78
Patient/client willingness to schedule sessions.	1	5	3.12	1.0

* Responses ranged from 0 = “Significantly worse with telehealth relative to in-person” to 5 = “Significantly better with telehealth relative to in-person.”
